# Fluid Structural Analysis of Human Cerebral Aneurysm Using Their Own Wall Mechanical Properties

**DOI:** 10.1155/2013/293128

**Published:** 2013-09-18

**Authors:** Alvaro Valencia, Patricio Burdiles, Miguel Ignat, Jorge Mura, Eduardo Bravo, Rodrigo Rivera, Juan Sordo

**Affiliations:** ^1^Department of Mechanical Engineering, Universidad de Chile, 8370448 Santiago, Chile; ^2^Institute of Neurosurgery Dr. Asenjo, 7500691 Santiago, Chile

## Abstract

Computational Structural Dynamics (CSD) simulations, Computational Fluid Dynamics (CFD) simulation, and Fluid Structure Interaction (FSI) simulations were carried out in an anatomically realistic model of a saccular cerebral aneurysm with the objective of quantifying the effects of type of simulation on principal fluid and solid mechanics results. Eight CSD simulations, one CFD simulation, and four FSI simulations were made. The results allowed the study of the influence of the type of material elements in the solid, the aneurism's wall thickness, and the type of simulation on the modeling of a human cerebral aneurysm. The simulations use their own wall mechanical properties of the aneurysm. The more complex simulation was the FSI simulation completely coupled with hyperelastic Mooney-Rivlin material, normal internal pressure, and normal variable thickness. The FSI simulation coupled in one direction using hyperelastic Mooney-Rivlin material, normal internal pressure, and normal variable thickness is the one that presents the most similar results with respect to the more complex FSI simulation, requiring one-fourth of the calculation time.

## 1. Introduction

An aneurysm is a localized dilation of the wall of an artery; it appears most frequently in the abdominal aorta or in the brain vasculature. Intracranial cerebral aneurysms are formed preferentially in abrupt curvatures or bifurcations of arteries belonging to the circle of Willis. In general, its geometry resembles a projecting dome on the wall of the artery. The formation of aneurysms represents the loss of the structural integrity of the wall, but the reasons for their formation and growth are still not clear. A subarachnoid hemorrhage due to the rupture of an intracranial aneurysm is a devastating event associated with large rates of morbidity and mortality. Approximately 12% of the patients die before receiving medical attention, 40% of hospitalized patients die within one month of the hemorrhage, and more than one-third of the patients that survive are left with an important neurological deficit [1].

A cerebral aneurysm wall has a thin tunica media, and the internal elastic lamina is normally severely fragmented. The aneurysm wall is generally composed of only intima and adventitia of layered collagen. Wall strength is related to both collagen fiber strength and orientation. The average break strength of aneurysm wall ranged from 0.7 MPa to 1.9 MPa [2]. Seshaiyer et al. [3] reported the mechanical properties of cerebral aneurysms. It was found that the lesions were stiffer than previously reported experimental data of Scott et al. [4] and Tóth et al. [5] and also stiffer as the mechanical properties of cerebral arteries reported by Monson et al. [6].

Wall shear stress (WSS) modulates endothelial cell remodelling via realignment and elongation. Consequently, fluid dynamics play important roles in the growth and rupture of cerebral aneurysms. Aneurysm rupture is related to a low level of WSS and therefore is associated with low flow conditions. The aneurysm region with low flow conditions is normally the fundus, [7]. High WSS is regarded as a major factor in the development and growth of cerebral aneurysms [8]. It is assumed that a WSS of approximately 2 Pa is suitable for maintaining the structure of the aneurysm wall, whereas a lower WSS results in the degeneration of endothelial cells via the apoptotic cell cycle [9].

This problem can be investigated using fluid structure interaction (FSI) simulations. However, few FSI investigations have been performed on patient specific aneurysms models [10, 11]. Torii et al. [10] investigated the FSI in cerebral aneurysms under normal and hypertensive blood pressures. Torii et al. [11] have calculated that the wall displacement in a model with variable wall thickness was 60% larger than that of the uniform wall model.

Our group has done FSI simulation work on the interaction between the wall of the aneurysm and the blood. The simulations have been made using computational geometries obtained from angiograms of real aneurysms. The results of the simulations indicate a relation between the shear stress on the wall caused by the blood flow and the zones of the aneurysm's wall that are subjected to greater stress [12]. All the simulations used mechanical behavior models of the aneurysm's wall developed by other authors [3, 4].

**Figure 1:**
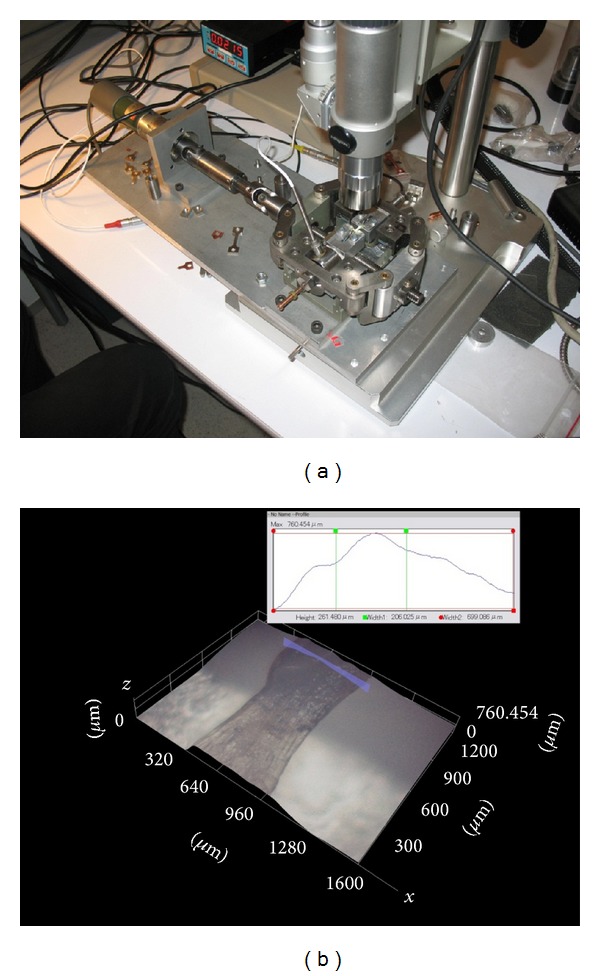
(a) Microtraction testing machine mounted under a microscope. (b) Measurement of the tissue thickness of the wall of aneurysm 1 [13].

As part of previous work done by our team [13], the mechanical behavior under traction of a cerebral aneurysm extracted from a patient was characterized, and a model of the mechanical behavior of the aneurysm's wall was developed.  Figure 1 shows the traction testing machine used and the measurement of the thickness of one of the samples. The present work consists in an advanced FSI modeling of the mechanical behavior of cerebral aneurysms, considering the background information on this type of behavior obtained in [13] in terms of experimental characterization and models. Only a single geometry will be used in the simulations, corresponding to the digitalization of the aneurysm used in [13] to create its model.

The simulations were made using the ADINA 8.8.0 commercial software. A five-parameter hyperelastic Mooney-Rivlin model was used to characterize the hyperelastic behavior of the aneurysm samples; this model with five parameter was found in [13] as the model that best fit the experimental data of the stress versus stretch ratio. The strain energy function *w* is given in the model by

(1) w(λ)=C10(I1−3)+C01(I2−3)+C11(I1−3)(I2−3)+C20(I1−3)2+C02(I2−3)2,

where *I*_1_ and *I*_2_ are the first and second strain invariants of the Cauchy-Green deformation tensor *C*_*ij*_,  *C*_*ij*_ = 2*ε*_*ij*_ + *δ*_*ij*_, where *δ*_*ij*_ is the Kronecker delta; *C*_10_, *C*_01_, *C*_11_, *C*_20_, and *C*_02_ are material constants, ADINA manual [14].

A linear elastic model was also used in which Young's modulus was obtained from the first two parameters of the Mooney-Rivlin model, following the recommendations of the ADINA manual [14]:

(2) $$\begin{array}{c} T=E*e, \end{array}$$

(3) $$\begin{array}{c} E=6*\left(C_{10}+C_{01}\right), \end{array}$$

where *T* is the stress, *e* is the engineering strain, and *E* is Young's modulus of the material.

**Figure 2:**
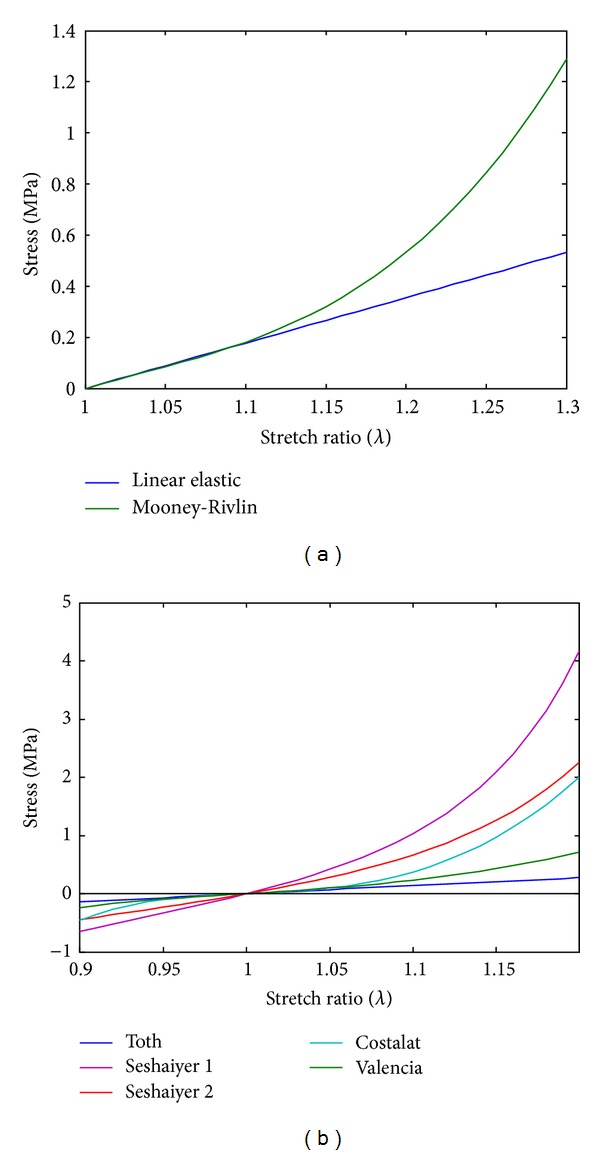
(a) Model of the wall material of the aneurysm fitted through Mooney-Rivlin and the linear elastic model by Contente [13]. (b) Hyperelastic models by Seshaiyer et al. [3], Töth et al, [5], Costalat and Sanchez [15] and Contente [13].

Table 1 shows the five parameters of the hyperelastic Mooney-Rivlin model and Young's modulus obtained from the experimental data from [13].  Figure 2(a) shows curves obtained from the experimental tests in [13], with the green curve corresponding to the fit made with the five-parameter Mooney-Rivlin model of the experimental data from [13] and the blue curve to that made with the linear elastic model approximation.  Figure 2(b) shows a comparison of the hyperelastic curve modeled through Mooney-Rivlin with other models found in the literature, Seshaiyer [3], Tóth et al. [5], and Costalat and Sanchez [15].

Costalat and Sanchez [15] have characterized the mechanical properties of sixteen intracranial aneurysms, for ruptured and unruptured samples. They have found a significant modification in biomechanical properties, the unruptured aneurysms were soft, they measured only to a strain of 10%, and therefore they do not report rupture strain and stress. Valencia's model falls within the ranges described by Costalat and Sanchez [15] and other authors [3, 5].

In this investigation, we present detailed numerical simulations of fluid and solid mechanics in an anatomically realistic cerebral aneurysm model using their own mechanical properties. The patient specific geometry was reconstructed from 3D rotational angiography image data. The predictions using CFD, CSD, and FSI in fluid and solid variables on the aneurysm 1 are compared. The effects of hypertensive pressure load, aneurysm wall thickness, and wall model are also reported. The principal goal of this work is to quantify the differences in prediction of fluid and solid variables between a time expensive complete FSI simulation with CSD simulations. In addition, preprocedural planning for cerebral aneurysm treatments will benefit from an accurate assessment of flow patterns, effective wall stress, and strain in the aneurysm, as presented here by means of computational simulations.

## 2. Methodology

### 2.1. Reconstruction

**Figure 3:**
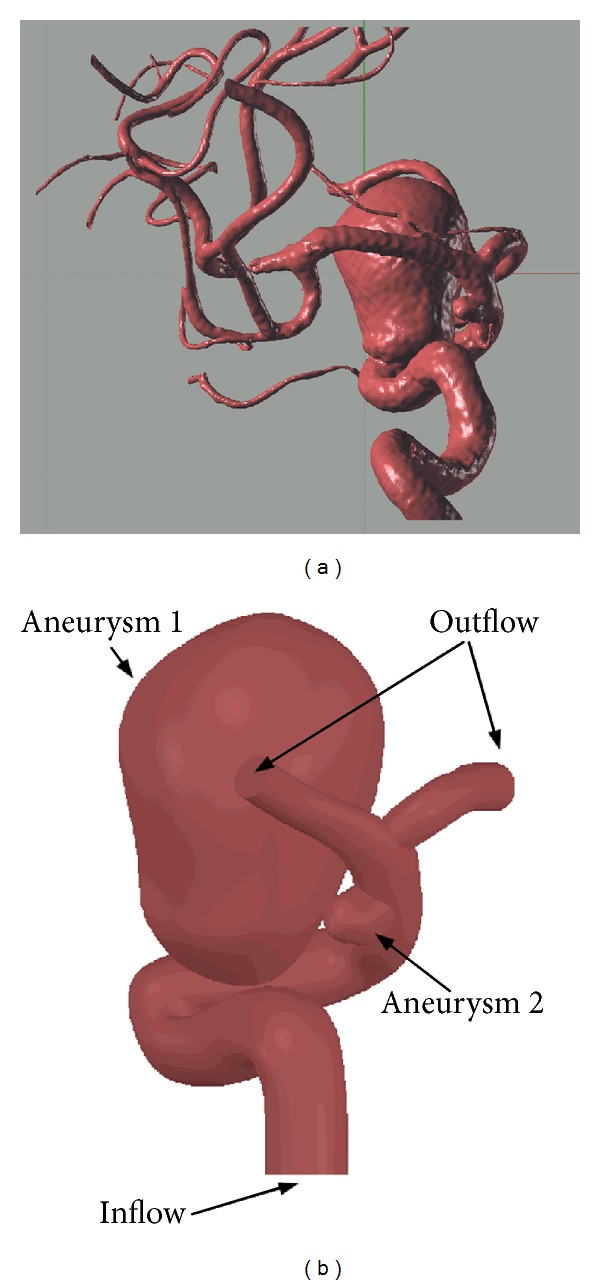
(a) Image obtained by angiography. (b) CAD obtained from the reconstruction of the original geometry.

**Figure 4:**
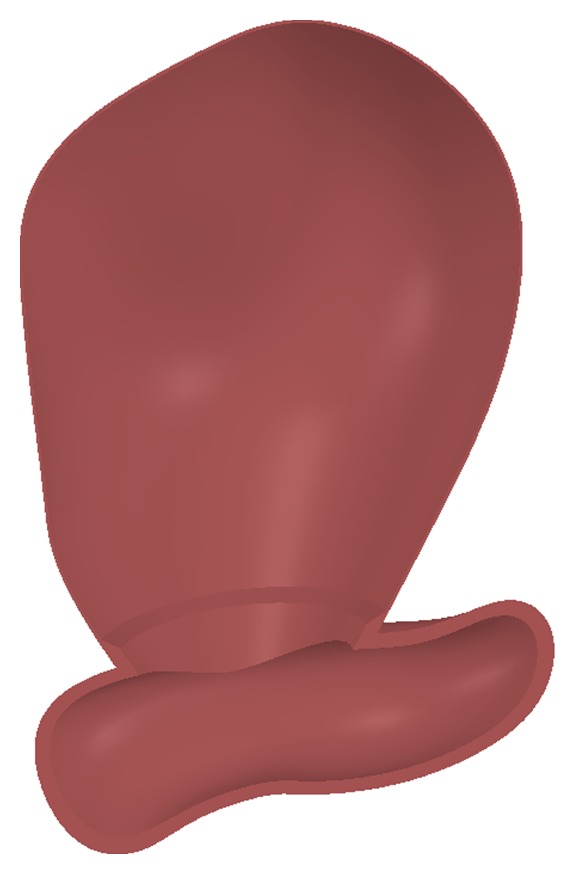
Detail of the thickness of the aneurysm, the artery, and the section by which they are joined.

To carry out the fluid dynamics simulations of real cases, it is necessary to generate 3D geometries of the aneurysms that can be used in the ADINA simulation software. For that purpose a case delivered by the Instituto de Neurocirugía Asenjo was available. The examinations were obtained with a Philips Integris Allura 3D Rotational Angiograph, and they deliver dimensional computational files in VRML format, as seen in Figure 3(a). Since the VRML files cannot be used directly with the ADINA simulation software, a meticulous reconstruction of each case must be made to obtain an adequate file to make the simulation. The method developed in [17] allows this task to be performed in a couple of hours. The reconstructed CAD model is shown in Figure 3(b). The difference between the original geometry and the reconstructed one is less than 5%. The aneurysms in the reconstructed geometry assigned the thickness measured in [13], while the artery assigned a theoretical thickness that corresponds to 10% of its diameter. The aneurysms are joined with the artery through a special section that varies from the thickness of the aneurysm to the thickness of the artery.  Figure 4 shows a detail of the variable thickness section.

The complete geometry is a single volume and is used for the CFD and FSI simulations. A hollow geometry is used to carry out the CSD simulations; it has artery thickness for the arteries, aneurysm thickness for the aneurysms, and a variable thickness for the junction between artery and aneurysm.  Table 2 summarizes the data of interest of the complete geometry, and Table 3 summarizes the relevant data of both aneurysms.

Table 4 shows the thicknesses of the aneurysm, the artery, and the section that joins both of them (aneurysm-artery junction or A-A junction). The thickness of the artery is considered to be 10% of its diameter. Some simulations use half the aneurysm thickness shown in Table 4, in order to see the results in a thinner aneurysm.

### 2.2. Constitutive Equations

The Navier-Stokes equations allow modeling fluids. These equations consider the conservation of mass (4) and the conservation of momentum (5) in the fluid:

(4) $$\begin{array}{c} \nabla \cdot v=0, \end{array}$$

(5) $$\begin{array}{c} \rho \frac{\partial v}{\partial t}+\nabla \cdot \left(\rho vv-\tau \right)=f^{B}, \end{array}$$

where *v* is the velocity, *ρ* is the fluid density, *τ* are the stress, and *f* are the body forces. 

The fluid is considered incompressible, and the flow is considered laminar. Carreau's model (6) is used to model the viscosity *μ*:

(6) $$\begin{array}{c} \mu \left(\dot{\gamma }\right)=\mu_{\infty }+\left(\mu_{0}-\mu_{\infty }\right){\left(1+A{\dot{\gamma }}^{2}\right)}^{n}. \end{array}$$

The Carreau blood model predicts decreasing viscosity at high strain, where *μ*_0_ and *μ*_*∞*_ are low and high shear rate asymptotic values and the parameters *A* and *n* control the transition region size. We have taken the values used in [16] as *μ*_*∞*_ = 0.00345 Ns/m^2^, *μ*_0_ = 0.056 Ns/m^2^, *k* = 10.976, and *m* = −0.3216. The density of blood was assumed to be constant *ρ* = 1050 kg/m^3^.

The wall of the aneurysm is considered to be a linear elastic material (7) or a hyperelastic material (8):

(7) $$\begin{array}{c} S=C\varepsilon , \end{array}$$

(8) $$\begin{array}{c} S=\frac{1}{2}\left(\frac{\partial W}{\partial \varepsilon }\right), \end{array}$$

where *S* is the second Piola-Kirchhoff stress, *ε* is the Green-Lagrange strain, and *W* is the strain energy of the material. 

### 2.3. Boundary Conditions

For the simulations an averaged blood pulse is used in this case. In the study, Valencia el al. [12] used one pulse for each patient. Although this allows a study closer to each patient's reality, it adds an important variable to be considered in the analysis of the results: the heart rate. In view of the large difference in heartbeat from one patient to another, it is not difficult to understand that this will influence the results, and when comparing one case with another, it will not be known exactly whether the differences found are due to differences in the heart rate or in the shape of the aneurysm. That is, why it was decided to use an averaged pulse in this case. This averaged pulse cannot be just anyone, because the pulse of a healthy person is different from that of a sick person, so an average pulse was obtained from color duplex Doppler images of 36 patients with cerebral aneurysms.

**Figure 5:**
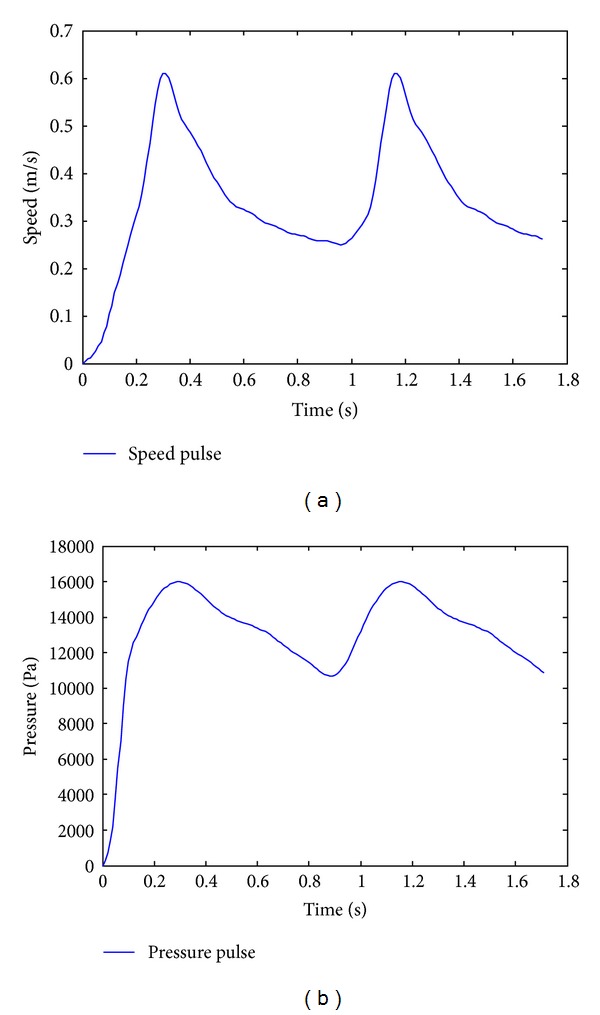
(a) Blood velocity pulse applied to the entrance section of the artery. (b) Blood pressure pulse applied to the outlet section of the artery.

To make a correct simulation of blood flow in the artery, it is necessary to use the correct velocity profile at the entrance. For pulsating flows in the arteries the classical parabolic profile is not sufficient to describe the flow of the velocity profile. Womersley's solution for a pulsating flow in a rigid tube has been used previously [16] with good results. To be able to implement Womersley's profile in the simulations, use will be made of the method developed in [16], which use the MATLAB calculation software to develop the profile and for later exporting to ADINA.  Figure 5(a) shows the average velocity profile in two pulses. Numericat tests performed by Valencia et al. in [12] have showed that after the second cardiac cycle, the results of velocity and pressure do not change in similar geometries of cerebral aneurysms, and the results of the first cycle are different because all variables start the simulations at zero. 

To simulate correctly the path of the blood through the cerebral arteries, an outlet condition must be used for the blood flow. If an outlet condition is not imposed, we would have a flow that is set free going out of the artery, therefore not representing reality, because the blood follows a closed path. To replicate this effect, an oscillating pressure resistance between 80 and 120 (mm Hg) in phase with the heartbeats is applied at the outlets of the section. This allows the replication of the closed circuit followed by the blood flow.  Figure 5(b) shows two pressure pulses. It is important to specify that this pressure is relative, not absolute.

The boundary conditions for CSD simulations imposed on the models was a time-dependent pressure on the inner wall representative for normal human pressure variation with a heart rate of 70 beats/min; see Figure 5(b). The effects of hypertension are reported using the temporal pressure variation between 180 mm Hg and 100 mm Hg. The outside pressure due the cerebrospinal fluid was considered constant as 3 (mm Hg) (400 (Pa)) was used by Valencia et al. [12]. The model was fixed on the inflow and outflow.

CSD simulations were made with a pressure pulse 200% greater than that shown in Figure 5(b), attempting to eliminate the increased internal pressure as a cause for the rupture of aneurysms. The cerebral arteries are not found in an empty environment, but they are rather immersed in cerebral fluid, which produces a constant external pressure that compresses the arteries radially.

On the FSI interface states that (i) displacements of the fluid and solid must be compatible, (ii) tractions at this boundary must be at equilibrium, and (iii) fluid obeys the no-slip condition. These conditions are given as follows:

(9) $$\begin{array}{c} \delta_{s}=\delta_{f}, \end{array}$$

(10) $$\begin{array}{c} \sigma_{s}\cdot {\hat{n}}_{s}=\sigma_{f}\cdot {\hat{n}}_{f}, \end{array}$$

(11) $$\begin{array}{c} u=u_{g}, \end{array}$$

where *δ*, *σ*, and $\hat{n}$ are displacement, stress tensor, and boundary normal with the subscripts *f* and *s* indicating a property of the fluid and solid, respectively. The condition of (10) does not require identical matching meshes between the two domains and instead supports the use of solution mapping to establish the equilibrium.

### 2.4. General Considerations of the Simulations

Thirteen simulations were made in this research, and their characteristics are summarized in Table 5. One CFD simulation (rigid walls), eight CSD simulations, and four FSI simulations were performed.

CSD simulations 2, 3, and 4 use linear elastic material, CSD simulations 5, 6, and 7 use hyperelastic Mooney-Rivlin material with the approximation of shell elements; in these simulation we investigate the wall thickness and hypertensive pressure effects. In the CSD simulations 8 and 9 we investigate the effect of 3D tetrahedral elements.

The FSI simulations 10 and 11 use linear elastic material; we investigate the effect of the method coupling, and in the FSI simulations 12 and 13 we investigate the effect of the method coupling using a hyperelastic Mooney-Rivlin material.

The coupling indicates how the solid and fluid models interact with one another. It is possible to have a completely coupled FSI simulation in which an equilibrium of strength and displacements between solid and fluid is sought for every time step, requiring several iterations between them and therefore longer calculation time. It is also possible to use FSI simulations coupled in one direction. The results obtained in the CFD simulation are applied to the solid at each time step, but in this case the solid does not affect the fluid. This decreases calculation time and the precision of the results. Mesh tests were made for the CSD and CFD simulations.

**Figure 6:**
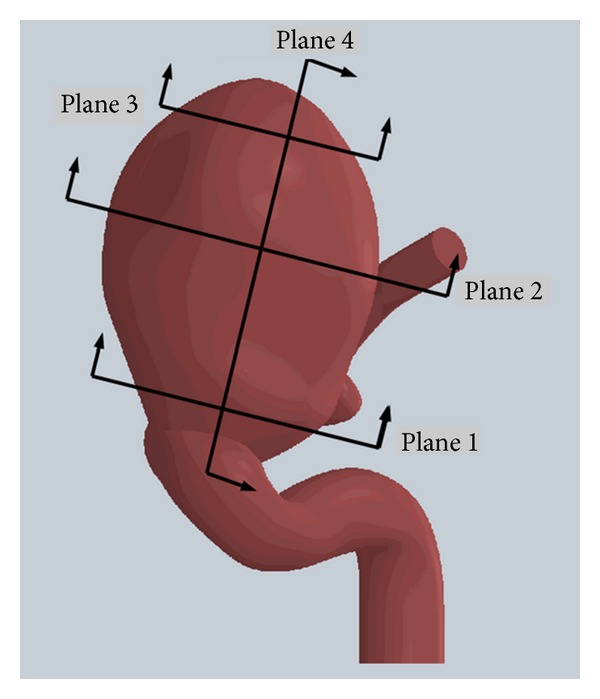
Control planes. Plane 1 or entrance plane, plane 2 or middle plane, plane 3 or upper plane, and plane 4 or transverse plane of aneurysm 1.

Figure 6 shows the control planes for aneurysm 1. Plane 4 is a transverse plane of aneurysm 1 perpendicular to its entrance area and parallel to the direction of flow in the artery. Planes 1, 2, and 3 are the entrance, middle, and upper planes of aneurysm 1, and they are parallel to the entrance area of aneurysm 1. The control points for each plane correspond to the element subjected to the maximum value of the variable in question.

**Figure 7:**
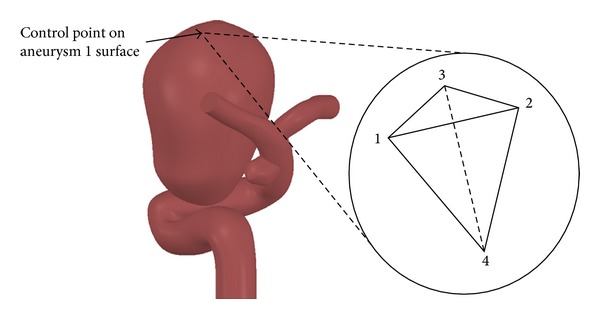
Control point in the fundus of aneurysm 1.

It is important to specify that the control element contains all the control points, so that the control element contains the data for the four nodes of the element. The datum for the control point is the node with the greater values, because in the case of the CFD the nodes that are on the wall (1, 2, and 3 in Figure 7) always have values equal to zero. In the case of CSD simulations with shell-type elements the element is triangular, so it only has nodes 1, 2, and 3 that are shown in Figure 7.

The rupture point in saccular cerebral aneurysm is located in the zone with maximum stress and deformation or in the aneurysm fundus; for this reason we define and study in details the results on the control point shown in Figure 7 related with the wall shear stress. Averaged values on the aneurysm surface do not provide useful information to study the growth and rupture of saccular aneurysms. We reported the local maximum values of several variables on aneurysm 1 and the temporal variations.

### 2.5. Numerical Method

 The CFD, FSI, and CSD models were solved by a commercial finite-element package ADINA 8.8.0. The Finite-Element Method (FEM) is used to solve the governing equations. The FEM discretizes the computational domain into finite elements that are interconnected by element nodal points. The fluid domain employs special Flow-Condition-Based-Interpolation (FCBI) tetrahedral elements. We have used the formulation with large displacements and small strains in the FSI calculation available in ADINA. The unstructured grids were composed of tetrahedral with four-node elements in the fluid and four-node isoparametric elements for the shell structure of the solid.

We have performed a sensitivity analysis of the mesh size in CFD and CSD simulations. Using a grid size with 110 elements/mm^3^, the difference on predictions of maximum wall shear stress at peak systole respect to a grid with 130 elements/mm^3^ were only 1%. For the CSD simulations we have used 1400 elements/mm^3^, and the difference with a grid of 1760 elements/mm^3^ on maximum displacement was only 1%. The mesh density used in each case is summerized in Table 6. For the integration of this time-dependent problem, we used the full implicit Euler method with a time step of Δ*t* = 0.01 s. The tolerance for all degrees of freedom was set to 0.001.

The workstation used to perform the simulations is based on an Intel Xeon dual core 64 bits processor of 3.0 Ghz clock speed, 8.0 Gb RAM memory. The simulation time for the FSI case based on 2 pulsatile flow cycles was around 29 CPU hours.

## 3. Results

The following results and discussions are based on aneurysm 1 due to this aneurysm that is the principal pathology in this patient. The aneurysm 2 is a part of the computational domain, ant it is located after the principal aneurysm, so that the secondary aneurysm does not affect the aneurysm 1 or principal pathology. The results of the aneurysm 2 are not discussed due to this an incipient geometrical change of the artery, and in this stage of development it cannot be classified as full saccular aneurysm. The patient is clinically treated due to the presence of the aneurysm 1 or pathology; the second aneurysm is in this case not part of the treatment. The aneurysm 2 is not analyzed.

The differences between the FSI simulations and the CFD and CSD simulations were studied with the purpose of observing the differences that are generated by not considering the interaction between the solid and the fluid.

The results are shown in two ways. The first corresponds to distribution graphics of simulation 12 only, because it was considered as the most complete. The second way of showing the results is by means of temporal graphics of significant variables on specific control points on aneurysm 1 considering several simulations. The significant variables in the fluid are pressure, wall shear stress, and velocity, while in the solid they are the displacement of the wall of the aneurysm 1, Von Mises effective stress, and first principal stress. The distribution graphics are shown at the times at which the significant variables are maximum or minimum. 

**Figure 8:**
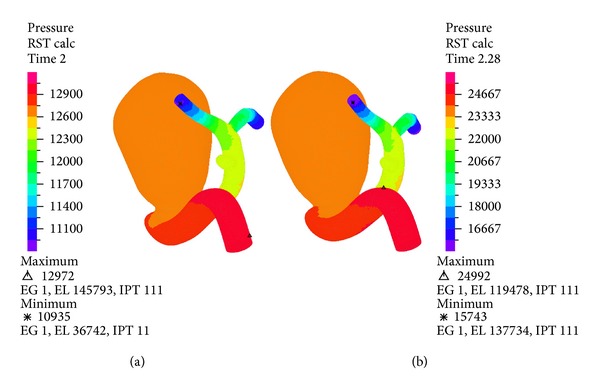
Simulation 12. Pressure distribution in the complete geometry during diastole (0.89 (s)) on the left and during systole (1.16 (s)) on the right.

Figure 8 shows the pressure distribution over the geometry for 0.92 (s) and 1.2 (s) for simulation 12. In both cases it can be seen that the pressure drops in the direction of the flow. The pressure drop is ~10 (kPa) during systole. The pressure on aneurysm is constant. 

**Figure 9:**
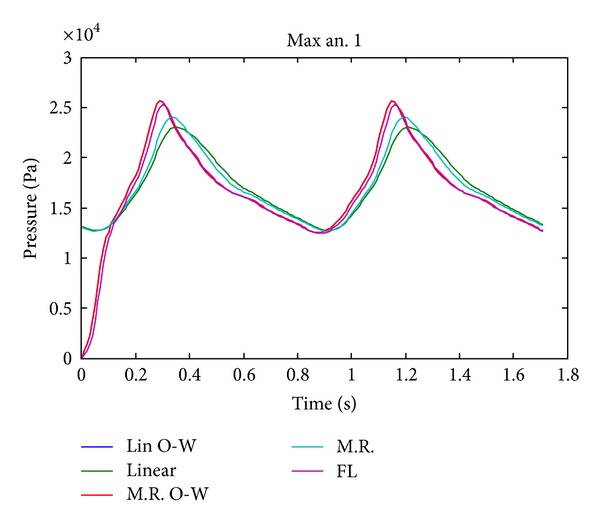
Temporal evolution of the maximum pressure in aneurysm 1 for simulation 1 (FL), simulation 10 (linear), simulation 11 (Lin O-W), simulation 12 (M.R), and simulation 13 (M.R O-W).

**Figure 10:**
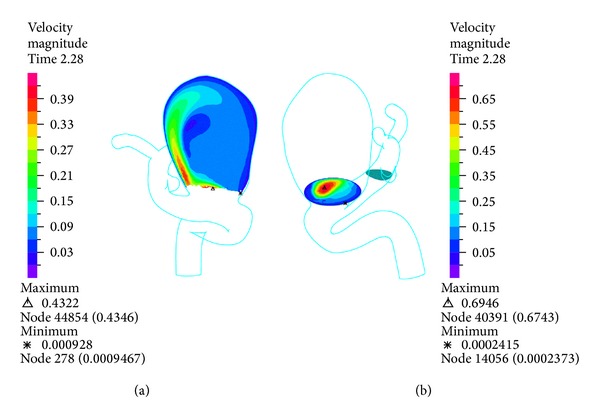
Simulation 12. Distribution of velocity at the transverse control (left) and entrance planes (right) for aneurysm 1 at peak systole (1.16 (s)).

**Figure 11:**
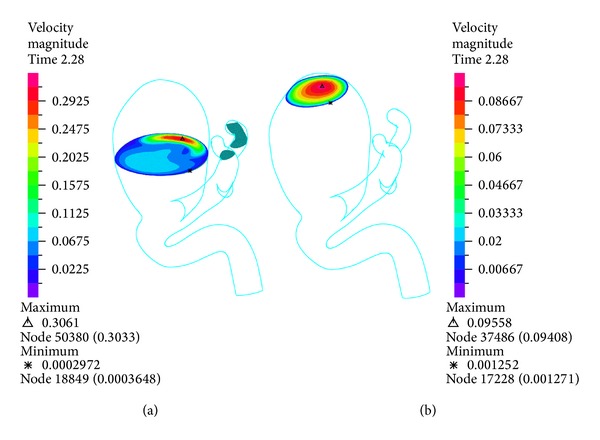
Simulation 12. Distribution of the velocity in the middle (left) and upper (right) control planes for aneurysm 1 at peak systole (1.16 (s)).

The temporal variation of pressure shown in Figure 9 shows that the differences on pressure between the CFD simulation 1 and FSI simulations 10, 11, 12, and 13 are low.  Figure 10 shows the velocity distribution for the transverse and entrance (plane 1) control planes, while Figure 11 shows the middle (plane 2) and upper (plane 3) planes of aneurysm 1 during systole. In the transverse plane (Figure 10) it is seen how the blood enters aneurysm 1 and goes to the left side of the wall due to the direction of the flow. In the entrance (Figure 10), middle and upper (Figure 11) planes, it is seen that the flow loses velocity as it starts recirculating around aneurysm 1. The maximum velocity at the upper of aneurysm 1 is one order of magnitude lower than the maximum velocity at the entrance to this aneurysm.

**Figure 12:**
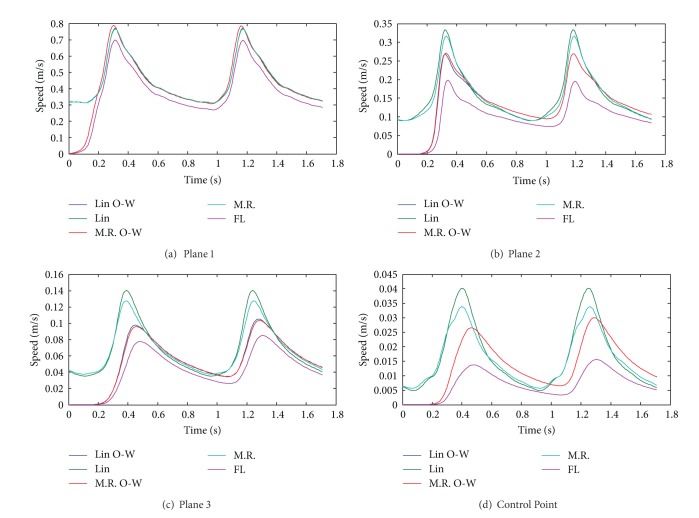
Temporal evolution of velocity in aneurysm 1 in the entrance plane (a), middle plane (b), upper plane (c), and control point (d) for simulation 1 (FL), simulation 10 (Lin), simulation 11 (Lin O-W), simulation 12 (M.R), and simulation 13 (M.R O-W).

The temporal evolution of the velocity in the aneurysm 1 in three planes and in the control point is shown in Figure 12; the effects of the model are very important in the magnitude of the velocity. The differences between CFD simulation 1 and FSI simulations 10, 11, 12, and 13 are relevant.

**Figure 13:**
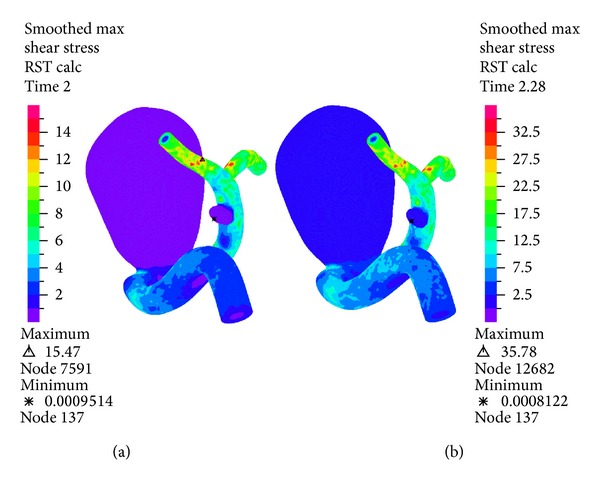
Simulation 12. Distribution of wall shear stress during diastole on the left and systole on the right.

**Figure 14:**
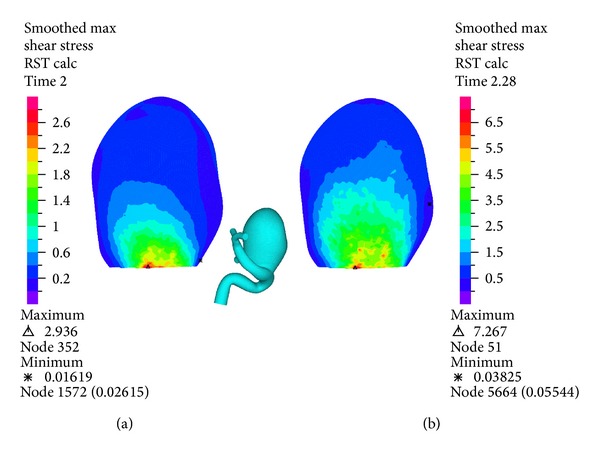
. Simulation 12. Distribution of wall shear stress during diastole on the left and systole on the right for aneurysm 1.

**Figure 15:**
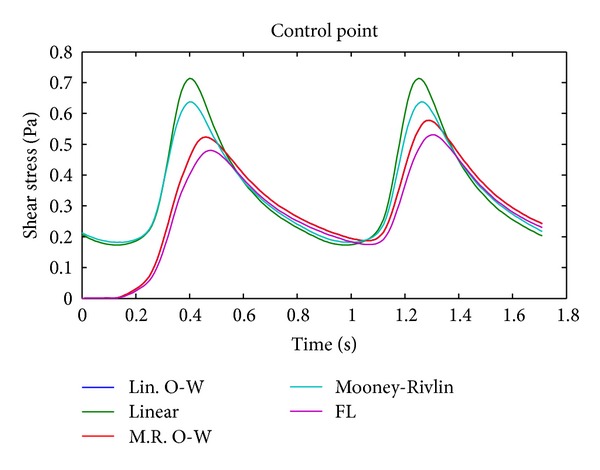
Temporal evolution of wall shear stress at the control point of aneurysm 1 for simulation 1 (FL), simulation 10 (linear), simulation 11 (Lin O-W), simulation 12 (Mooney-Rivlin), and simulation 13 (M.R O-W).

Figure 13 shows the wall shear stress distribution during diastole and systole in the complete geometry. It is seen that both aneurysms present lower wall shear stress compared to the rest of the geometry.  Figure 14 shows in detail the wall shear stress in aneurysm 1 with other color map ranges. The greatest wall shear stress is concentrated on the wall where the blood flow enters the aneurysm. The temporal evolution of wall shear stress at the control point of aneurysm 1 is shown in Figure 15; the effects of the model are very important in the wall shear stress. The FSI simulations 12 and 13 using Mooney-Rivlin show similar values of wall shear stress, except at systole, and the CFD simulation 1 shows low wall sheart stress. 

**Figure 16:**
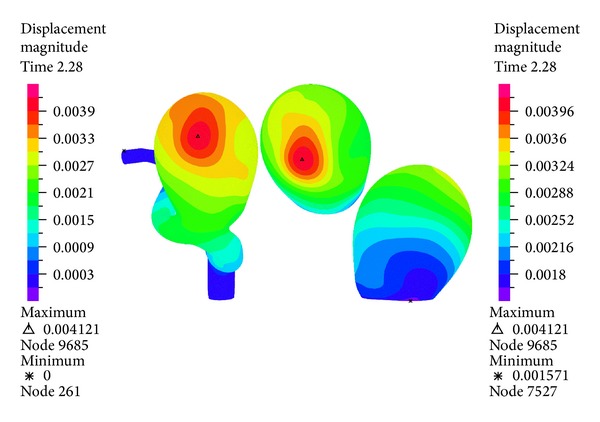
Simulation 12. Displacement distribution on the wall of aneurysm 1 at peak systole (1.16 (s)).

Figure 16 shows the displacement distribution for the wall of the geometry during systole. It can be seen that the maximum displacement occurs on the left side of aneurysm 1 with respect to the entrance-outlet direction of the blood flow. The maximum displacement is 4.1 (mm), which is important considering the size of the aneurysm 1.

**Figure 17:**
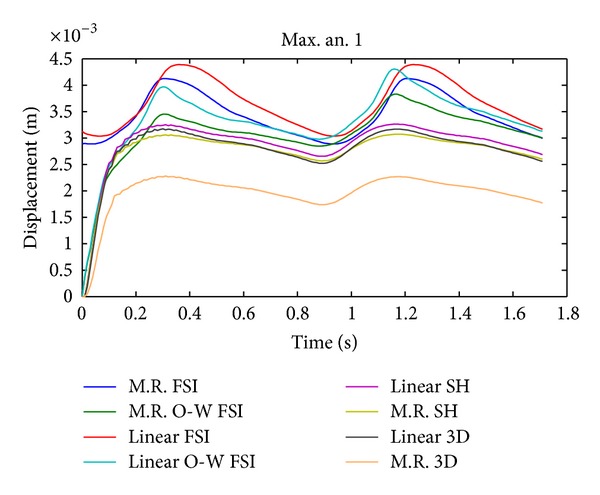
Temporal evolution of maximum displacement in aneurysm 1 for simulation 2 (linear SH), simulation 5 (M.R SH), simulation 8 (linear 3D), simulation 9 (M.R 3D), simulation 10 (linear FSI), simulation 11 (linear O-W FSI), simulation 12 (M.R FSI), and simulation 13 (M.R O-W FSI).

The temporal evolution of maximum displacement in aneurysm 1 for CSD simulations 2, 5, 8, and 9 and FSI simulations 10, 11, 12, and 13 is shown in Figure 17; the CSD predicts lower wall displacements compared with the FSI results. 

**Figure 18:**
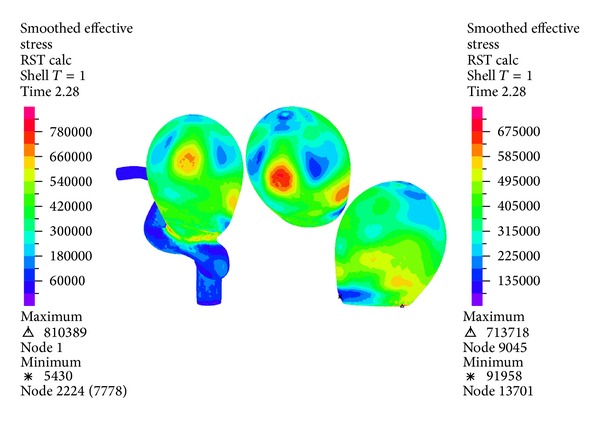
Simulation 12. Distribution of the effective Von Mises stress on the wall of aneurysm 1 during systole (1.16 (s)).

Figure 18 shows the distribution of effective Von Mises stress on the wall of the artery and of aneurysm 1. The high stress zone occurs near the dome of aneurysm 1. In aneurysm 1 the maximum stress occurs in the same zone as the maximum displacement. The overall maximum is 100 (kPa) higher than the maximum stress in aneurysm 1, which is 713 (kPa).

**Figure 19:**
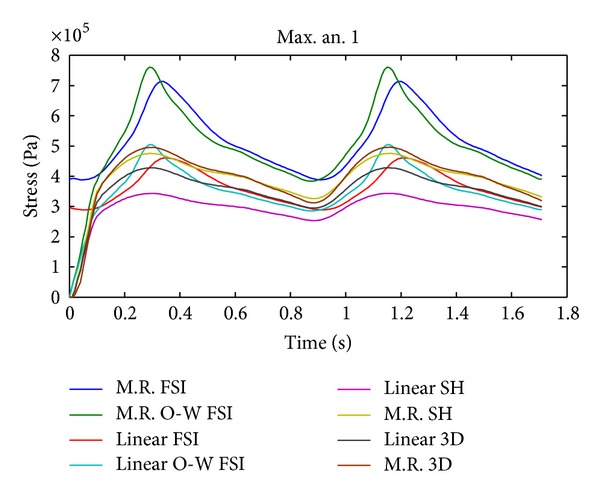
Temporal evolution of the effective maximum Von Mises stress in aneurysm 1 for simulation 2 (linear SH), simulation 5 (M.R SH), simulation 8 (linear 3D), simulation 9 (M.R 3D), simulation 10 (linear FSI), simulation 11 (linear O-W FSI), simulation 12 (M.R FSI), and simulation 13 (M.R O-W FSI).

The temporal evolution of the effective maximum Von Mises stress in aneurysm 1 for CSD simulations 2, 5, 8, and 9 and FSI simulations 10, 11, 12, and 13 is shown in Figure 19; the CSD predicts lower Von Mises stress compared with the results of FSI models with Money-Rivlin. 

**Figure 20:**
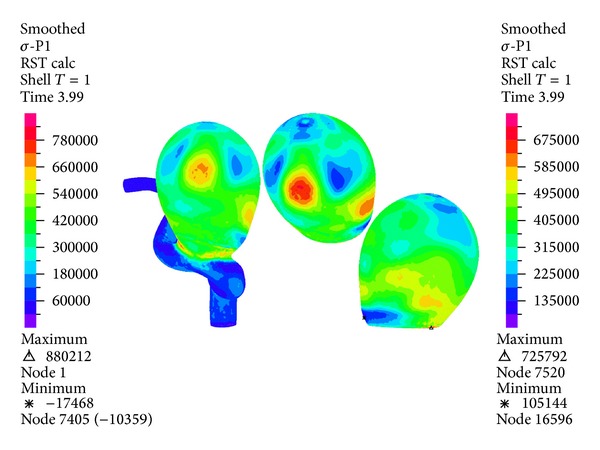
Simulation 12. Distribution of the first principal stress on the wall of aneurysm 1 during systole (1.16 (s)).

Figure 20 shows the distribution of the first principal stress on the wall of the artery and of aneurysm 1 during systole. Again, the maximum stress for the whole geometry occurs in the neck of aneurysm 1 and is 150 (kPa) higher than the first principal maximum stress of aneurysm 1, which is 725 (kPa). For aneurysm 1 the area on which the first principal maximum stress is concentrated coincides with the area on which the maximum effective Von Mises stress and the maximum displacement are concentrated. 

**Figure 21:**
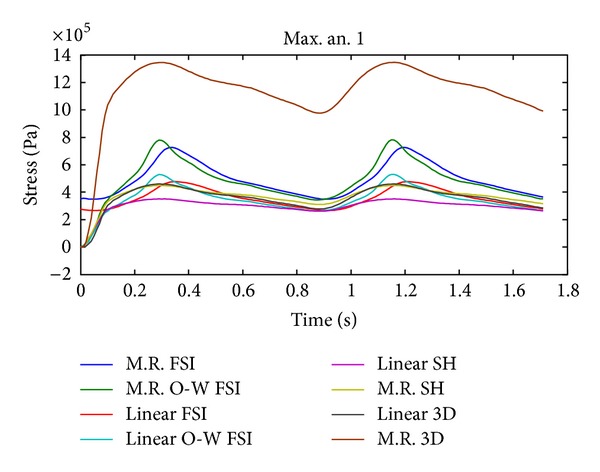
Temporal evolution of the first principal maximum stress in aneurysm 1 for simulation 2 (linear SH), simulation 5 (M.R SH), simulation 8 (linear 3D), simulation 9 (M.R 3D), simulation 10 (linear FSI), simulation 11 (linear O-W FSI), simulation 12 (M.R FSI), and simulation 13 (M.R O-W FSI).

The temporal evolution of the first principal maximum stress in aneurysm 1 for CSD simulations 2, 5, 8, and 9 and FSI simulations 10, 11, 12, and 13 is shown in Figure 21; the CSD predicts similar stress compared with the results of FSI models except for the simulation 9.

**Figure 22:**
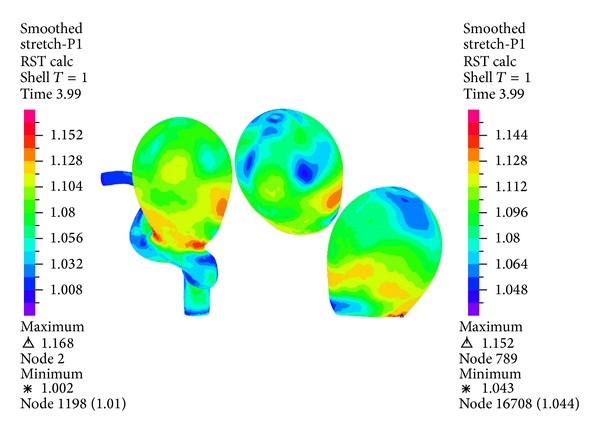
Simulation 12. Distribution of the first principal stretching in the wall of aneurysm 1 at peak systole (1.16 (s)).

**Figure 23:**
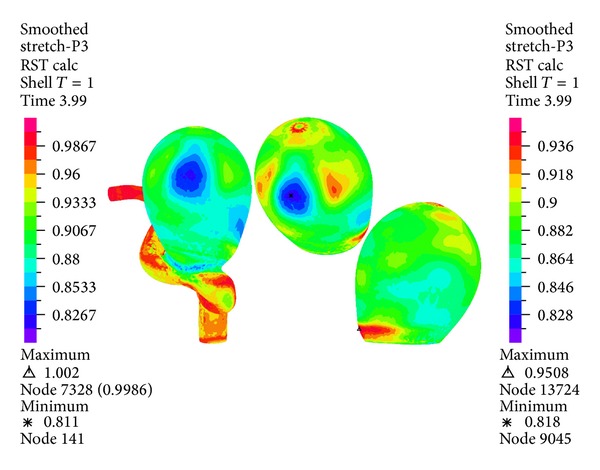
Simulation 12. Distribution of the third principal stretching in the wall of aneurysm 1 under systole (1.16 (s)).

Figure 22 shows the first principal stretching for the geometry and aneurysm 1 under systole. For the geometry the maximum stretching is concentrated in the neck of aneurysm 1. It is seen that the high displacement zone appears in the equator of aneurysm 1 (see Figure 16), and the maximum stress zone does coincide in this case. The first principal deformation is positive, so there is traction.  Figure 23 shows the third principal stretch for the geometry and aneurysm 1 under systole. It is seen that the minimum value for the third principal stretch in the aneurysm 1 has a value of 0.95. Since the stretching is smaller than 1, we have compressive stress.

The FSI simulation 12 with complete coupling, with normal pressure and tetrahedral 3D elements, is considered the most complete simulation and is taken in the discussion as reference to compare results of the solid and fluid dynamics. The values presented in Tables 7, 8, 9, 10, 11, and 12 are for the larger aneurysm (aneurysm 1) due this is the relevant pathology in this investigation. The values in Tables 7 and 11 are the maximum at peak systole of the second cardiac cycle.  Table 7 shows solid results, and Table 11 shows fluid results. Tables 8, 9, 10, and 12 present percentage differences.

## 4. Discussion

The discussion of principal results is limited to the values presented in Tables 7 and 11 because they include the most relevant fluid and solid variables of the aneurysm 1 at peak systole. 

Using Table 7 can report the influence of blood hypertension, wall thickness, linear elastic and hyperelastic Mooney-Rivlin wall model, shell or 3D tetrahedral elements, differences between CSD and FSI models, and effect of FSI coupling. Differences between CFD and FSI models for fluid variables are reported in Table 11.

The comparisons are mostly presented as percentage with respect to the results of FSI simulation 12 considered as more complete simulation, unless otherwise stated. The comparisons are made at the systolic time to report the maximum values of each variable. Tables 8, 9, 10, and 12 show percentage differences.

### 4.1. Solid

Effective maximum stresses between 344 (kPa) and 785 (kPa) were obtained for simulations with normal internal pressure; this values are within the ranges described in the literature [18].  Table 7 shows the values at peak systole for selected variables for simulations 2 to 13.  Table 8 shows the differences between CSD simulations (simulations 2, 5, 8, and 9) and the solid part of the FSI simulations (simulations 10, 11, and 13) with simulation 12.

Figures 16, 18, 20, 22, and 23 show the critical zones for each variable in the geometry for simulation 12. The displacement, the effective Von Mises stress, and the third principal deformation are concentrated on the side of aneurysm 1, near the dome. The first principal stress and the first principal deformation are concentrated on the base, where the aneurysm joins the artery.

#### 4.1.1. Cell Type

In Figures 17, 19, and 21 it is seen that the CSD simulations with 3D elements and shell-type elements present important differences with respect to FSI simulation 12. The use of shell-type elements in the CSD simulations underestimates the obtained stress, compared with CSD simulations with solid tetrahedral 3D elements. At this point it is necessary to consider if a mesh composed by 3D elements is adequate for simulating thin-shell models. The simulations with shell-type elements show the same critical regions as the simulations with 3D elements.

Using the CSD results, the use of shell-type elements can be evaluated by comparing the CSD simulations 2 versus 8 for elastic material and the CSD simulations 5 versus 9 for Mooney-Rivlin material in Table 7. The difference in effective stress is 20% for the elastic material and 4% for the Mooney-Rivlin material compared with the 3D tetrahedral elements.

#### 4.1.2. Hypertension

The effect of hypertension can be reported by comparing the CSD simulations 2 and 3 for elastic material and the CSD simulations 5 and 6 for Mooney-Rivlin material. The increase in effective stress is 167% for the elastic material and 195% for the Mooney Rivlin material. The differences between the 3D solid model and the shell approach in CSD simulations can be seen comparing the simulation 5 and the simulation 9; the differences on maximum effective stress are only 4%. The differences due the hypertension are larger, and this validates the comparison using shell elements. The hypertension patient condition is one of the most relevant parameter. Pressure cannot be discarded as one of the most important causes of rupture of the aneurysm, given the linear relation that it has with the effective stress. The simulations with hypertensive internal pressure show very high maximum effective stresses, in agreement with an internal pressure higher than normal.

#### 4.1.3. Wall Thickness

In the simulations with normal thickness (simulations 2 and 5) it is seen that the critical zones are located in the neck and the dome of aneurysm 1. This agrees with what has been found in [12], who used the artery thickness for the whole geometry. It should be recalled that, in the present study, the thickness measured for the aneurysm is very close to the theoretical value for the artery. 

In the simulations with half the normal thickness (simulations 4 and 7) it is seen that the critical zones are localized in the dome of aneurysm 1. This agrees with what is reported in [16], who used an aneurysm thickness one order of magnitude less than that of the artery. In Tables 9 and 10 it can be seen that the simulations with half the normal thickness show displacements that are between 13% and 21% greater than the simulations with normal thickness (simulations 2 and 5). Furthermore, the stresses are 67%, and the deformations are 43% greater than those delivered by the simulations with normal thickness. All this could be expected, since the geometry in simulations 4 and 7 has a smaller thickness that must support the same loads, so the internal stress, the deformations, and the displacements will be greater. 

#### 4.1.4. Simulation Type

The CSD simulation 9 underestimates the maximum effective stress by 31%, the maximum of 1st principal deformations by 7%, and the maximum displacements by 44% with respect to the FSI simulation 12 on the aneurysm 1, but they can deliver the regions where the most important stresses and deformations are localized, which are very close to the zones determined in the FSI simulations, so the CSD simulations can be useful at the time of identifying risk zones.

#### 4.1.5. Coupling

FSI simulations 11 and 13 coupled in only one direction present differences of 10% and 7% in the maximum effective stress with respect to FSI simulations completely coupled with similar material (comparing simulation 11 with 10 and simulation 13 with simulation 12); see Table 7. In aneurysm 1 it is seen that the high stress zones coincide with high deformation zones. FSI simulations show differences for all the studied values when the simulation material is modified. The simulation 10 with linear elastic material overestimates the displacement by 7% and the 1st principal deformation by 33%, while it underestimates the effective stress by 35% with respect to the FSI simulation 12 with hyperelastic material. FSI simulation 13 coupled in one direction underestimates the maximum displacement by 7%, while it overestimates the effective stress by 7% and the 1st principal deformation by 7% with respect to the completely coupled FSI simulation 12. There is a relation between the effective stress on the wall of aneurysm 1 and the internal pressure in that aneurysm.

It is interesting to note that the displacement and stress curves (Figures 17, 19, and 21) for the completely coupled simulations are delayed with respect to the pure and coupled solid simulations in one direction. Figure 9 shows that the pressure curves within the geometry undergo the same delay as that of the curves in the previously mentioned figures, showing that this is caused by the fluid's pressure.

#### 4.1.6. Material

In Table 8 it can be seen that the FSI simulation with linear elastic material (simulations 10) overestimates the displacement by 7% and the deformations by 33%, while they underestimate the stress by 35% with respect to the FSI simulation with hyperelastic material (simulations 12). 

In Table 7 it is clearly seen that the simulations with Mooney-Rivlin material (simulations 5, 9, 12, and 13) present greater stress and lower deformations than the simulations with linear elastic material (simulations 2, 8, 10, and 11). This difference is general and does not depend on the kind of element or coupling used. The explanation of this difference has to do with the shapes of the stress-deformation curves of both materials. Using a linear elastic model instead of a hyperelastic material leads to an underestimation of the stress by an average of 29%. 

### 4.2. Fluid

Table 11 shows the values at peak systole of the studied variables for simulations 1, 10, 11, 12, and 13.  Table 12 shows the differences for the principal variables in the fluid for simulations 1, 10, 11, and 13, compared with the fluid part of simulation 12 (FSI, Mooney-Rivlin, completely coupled). It is seen in Table 11 that the pressures in aneurysm 1 are very similar for simulations 1, 10, 11, 12, and 13 and that the differences are less than 7%. In Table 12 it can be seen that the pressure shows the smallest difference between the models that are being compared, followed by shear stress, and finally the velocity shows the largest difference. It is also found that the highest velocity differences occur on control point in aneurysm 1, as shown in Figure 12.

#### 4.2.1. Simulation Type

The CFD simulation (simulation 1) underestimates the velocity inside the aneurysm, as seen in Figure 12. It also underestimates the wall shear stress value at the control point. Figure 15 shows these differences graphically.

The CFD simulation tends to underestimate the velocity in aneurysm 1, and the difference increases as the fluid approaches the dome of aneurysm 1. The CFD simulation tends to overestimate the maximum pressure on 5% with respect to the completely coupled FSI simulations at peak systole; see Table 8. The CFD simulation delivers a wall shear stress at the control point of aneurysm 1 17% lower than that seen in completely coupled FSI simulation 12. Finally, the CFD simulation underestimates the flow velocity in the upper plane on aneurysm 1 by 31% with respect to the completely coupled FSI simulation 12. 

The explanation for these differences could be the movement of the aneurysm's wall. When it is displaced, it favors the flow, causing a smaller decrease of the velocity compared to the solid wall model. The higher velocities in the aneurysm explain why a greater difference is seen in the wall shear stress at the control point than in the wall shear stress at the maximum point for aneurysm 1.

#### 4.2.2. Coupling

The simulations coupled in one direction (simulations 11 and 13) overestimate the pressure values (Figure 9) and underestimate the wall shear stress (Figure 15), and they show lower velocities in aneurysm 1 (Figure 12) regarding simulations 10 and 12 which are fully coupled.  Table 12 shows that the maximum internal pressure delivered by the FSI simulation coupled in one direction is 6.8% higher than that shown by completely coupled simulations. Maximum wall shear stresses varying between 7.3 (Pa) and 8.3 (Pa) were obtained in aneurysm 1. Wall shear stress outside and at the entrance of the aneurysm is between 8.5% and 14.2% greater than that seen in completely coupled simulations, but the wall shear stress at the bottom of the aneurysm in the latter is 10.9%. The velocities at the entrance plane in aneurysm 1 are slightly faster for the simulations coupled in one direction, between 2.3% and 2.7%, but at the control point of aneurysm 1, they are between 14.4% and 15.4% faster for the completely coupled simulations.

The completely coupled simulations (simulations 10 and 12) present well-defined vortices in the middle plane, on both sides of the faster flow. In the simulations coupled in only one direction (simulations 11 and 13) a single vortex appears, to the left of the flow and not as well defined as those that are seen in the cases mentioned previously. The completely coupled simulations search a convergence between the results of the solids and fluids, leading to a more ordered flow than that seen in the case coupled in one direction.

Figure 12 shows the maximum velocity at the selected control planes. It is interesting to note that the simulations coupled in one direction and the CFD simulation have slower velocities than the completely coupled simulations, and the velocity curves are more delayed for the former. On the other hand, Figure 9 shows that the pressure is delayed and is lower than that of the uncoupled and CFD simulations. Clearly, the cause of these phenomena is the complete coupling, because the simulations that were made with that characteristic are the ones that show high and more advanced velocities in time, together with delayed and lower pressures. The reason for which complete coupling allows higher velocities and lower pressures is the velocity and momentum of the fluid, acting together with the displacement of the arterial wall.

The higher velocity of the completely coupled simulations accounts for the grater wall shear stress (Figure 15). The displacement of the arterial wall may be the cause for the lower pressures seen in these simulations. The distribution graphs for the pressure and wall shear stress in the CFD and FSI simulations show that the CFD simulation succeeds in determining the critical zones for the reported variables. It should be mentioned that aneurysm 1 shows minimum wall shear stresses in the order of 0.02 (Pa). The lower shear stress in aneurysm 1 is accounted for by the lower velocities achieved. Such low shear stress values represent an important rupture risk. 

#### 4.2.3. Material

Comparing the difference generated by the wall material of the geometry, it can be seen that the simulation 10 with linear elastic material tends to overestimate the wall shear stress, but it must be pointed out that the differences is only 11% with respect to simulation 12 at peak systole; see Table 8. In the aneurysm 1, it is seen that simulation 10 overestimates the velocity at the upper plane only in 8% with respect to simulation 12.

### 4.3. General Aspects

The patient has two aneurysms, but the aneurysm 2 is small, and it was clinically not classified as saccular aneurysm; therefore the results of aneurysm 1 were not compared with the aneurysm 2, and results for aneurysm 2 are not reported. The patient was diagnosed by the large aneurysm 1. A comparison of the FSI results of two saccular aneurysms of similar size in one patient is reported in detail in [16]. 

The present investigation is subject to some important limitations. There was no direct interaction of the computational geometries with the surrounding vasculature and cerebral tissue, since their inclusion would demand additional computational resources, and the fluid/solid boundary conditions required for such interactions are not well known. In addition, the effects of the anisotropy of the constitutive material would be necessary to be included in the future as a validation strategy for our computational modeling predictions. The use of a Windkessel model for modeling outflow conditions is believed to yield patient specific pressure variations for the vascular geometry. Finally the error of experimental data affects the absolute values reported in the simulation and the use of this as predictable medical tools for clinical applications.

## 5. Conclusions

The FSI simulations coupled in one direction as well as the CFD simulation are capable of showing the critical pressure and wall shear stress regions. In general, higher stresses are obtained when hyperelastic material is used, compared to linear elastic material. Simulations with solid 3D elements show greater stresses than those of the simulations with shell-type elements. The internal pressure in the artery and the thickness of the aneurysm are directly related to the stresses generated on the aneurysm's wall. The completely coupled FSI simulation with material fitted for Mooney-Rivlin delivers results for the Von Mises stress almost twice as large as those obtained in pure CSD simulations, but even so, the latter succeed in showing the critical zones in the aneurysm. FSI simulations coupled in one direction with material fitted for Mooney-Rivlin deliver results with less than 10% error in a reasonable calculation time.

## Figures and Tables

**Table 1:** Fitting coefficients through five-parameter Mooney-Rivlin model and linear elastic model.

Model	Coefficients (MPa)
Mooney Rivlin, five-parameters	*C*_10_ = 0.3848, *C*_01_ = −0.0891, *C*_11_ = 0.5118, *C*_20_ = 0.5109, *C*_02_ = 0.4912
Linear elastic	*E* = 1.7742

**Table 2:** Relevant dimensions for the reconstructed geometry.

Artery	
Entrance diameter (mm)	4.98
Entrance area (mm^2^)	19.46
Total CAD volume (mm^3^)	3862

**Table 3:** Relevant aneurysm dimensions.

Aneurysm	Neck diameter (mm)	Width (mm)	Length (mm)
1 (larger)	9.2	16.1	20.8
2 (smaller)	3.42	3.33	4.39

**Table 4:** Thickness of the aneurysm, the artery, and the junction between them.

Section	Thickness (mm)
Aneurysm	0.35
A-A junction	0.38
Artery	0.4

**Table 5:** Numbering and characterization of the simulations made.

Simulation	Type	Elements	Pressure	Thickness aneurysm	Material	Coupling
1	CFD	Tetrahedral	Normal	—	—	—
2	CSD	Shell	Normal	Normal	L.E	—
3	CSD	Shell	H.T	Normal	L.E	—
4	CSD	Shell	Normal	1/2	L.E	—
5	CSD	Shell	Normal	Normal	M.R	—
6	CSD	Shell	H.T	Normal	M.R	—
7	CSD	Shell	Normal	1/2	M.R	—
8	CSD	Tetrahedral	Normal	Normal	L.E	—
9	CSD	Tetrahedral	Normal	Normal	M.R	—
10	FSI	Tetrahedral	Normal	Normal	L.E	Complete
11	FSI	Tetrahedral	Normal	Normal	L.E	One direction
12	FSI	Tetrahedral	Normal	Normal	M.R	Complete
13	FSI	Tetrahedral	Normal	Normal	M.R	One direction

H.T is hypertension blood pressure ranged between 13200 Pa and 23800 Pa. Normal pressure is the blood pressure pulse shown in Figure 5(b), L.E. is the linear elastic wall model, and M.R. is the hyperelastic Mooney-Rivlin wall model.

**Table 6:** Mesh density and characteristics of the elements.

Element	Geometry	Nodes/element	Element size (mm)	Mesh density (elements/mm^3^)
3D solid	Tetrahedral	4	0.2	1400
3D fluid	Tetrahedral	4	0.33	110
Shell	Triangular	3	0.33	25

**Table 7:** Maximum displacement, effective stress, principal stress, and deformations for the CSD and FSI simulations at peak systole (1.16 (s)) for aneurysm 1.

Simulation	Displacement mm	Effective stress kPa	1st principal stress kPa	1st principal deformation. —	3rd principal deformation. —
2	3.3	344	351	0.14	−0.18
3	6.7	917	996	0.40	−0.48
4	4	551	594	0.22	−0.29
5	3.1	476	449	0.12	−0.15
6	5.3	1403	1565	0.24	−0.23
7	3.5	785	786	0.16	−0.18
8	3.2	428	460	0.17	−0.18
9	2.3	496	1347	0.14	−0.15
10	4.4	461	477	0.20	−0.24
11	4.3	505	529	0.22	−0.26
12	4.1	714	726	0.15	−0.18
13	3.8	761	781	0.16	−0.19

**Table 8:** Percentage difference between peak values for maximum deformation, effective stress, principal stress, and deformations for simulations 2, 5, 8, 9, 10, 11, and 13 regarding simulation 12 for aneurysm 1.

Simulation	Deformation %	Effective stress %	1st principal stress %	1st principal deformation. %	3rd principal deformation. %
2	19.5	51.8	51.7	6.6	0
5	24.4	33.3	38.2	20	16.6
8	22.0	40.0	36.6	13.3	0
9	43.9	30.5	85.5	6.6	16.6
10	7.3	35.4	34.3	33.3	33.3
11	4.9	29.3	27.1	46.7	44.4
13	7.3	6.6	7.6	6.7	5.6

**Table 9:** Percentage difference between peak values for maximum deformation, effective stress, principal stress, and deformations for simulations 3 and 4 regarding simulation 2 for aneurysm 1.

Simulation	Deformation %	Effective stress %	1st principal stress %	1st principal deformation. %	3rd principal deformation. %
3	103.0	166.6	138.8	185.7	166.7
4	21.2	60.2	69.2	57.1	61.1

**Table 10:** Percentage difference between peak values for maximum deformation, effective stress, principal stress, and deformations for simulations 6 and 7 regarding simulation 5 for aneurysm 1.

Simulation	Deformation %	Effective stress %	1st principal stress %	1st principal deformation. %	3rd principal deformation. %
6	71.0	194.7	248.6	100	53.3
7	12.9	64.9	75.1	33.3	20.0

**Table 11:** Maximum pressure on aneurysm 1, velocities, and wall shear stress on control point for CFD and FSI simulations at peak systole (1.16 (s)) for aneurysm 1.

Simulation	Pressure aneurysm 1 (Pa)	Velocity entrance plane aneurysm 1 (m/s)	Velocity upper plane aneurysm 1 (m/s)	Wall shear stress control point (Pa)
1	25300	0.70	0.09	0.53
10	23039	0.77	0.14	0.71
11	25660	0.79	0.11	0.57
12	24028	0.77	0.13	0.64
13	25660	0.79	0.11	0.57

**Table 12:** Percentage difference between peak values for maximum pressure, velocities, and wall shear stress for simulations 1, 10, 11, and 13 regarding simulation 12 on aneurysm 1.

Simulation	Pressure aneurysm 1 (%)	Velocity entrance plane aneurysm 1 (%)	Velocity upper plane aneurysm 1 (%)	Wall shear stress control point (%)
1	5.3	9.1	30.8	17.2
10	4.1	0.4	7.7	10.9
11	6.8	2.6	15.4	10.9
13	6.8	2.7	15.4	10.9
